# Preventive Inositol Hexaphosphate Extracted from Rice Bran Inhibits Colorectal Cancer through Involvement of Wnt/****β****-Catenin and COX-2 Pathways

**DOI:** 10.1155/2013/681027

**Published:** 2013-10-24

**Authors:** Nurul Husna Shafie, Norhaizan Mohd Esa, Hairuszah Ithnin, Abdah Md Akim, Norazalina Saad, Ashok Kumar Pandurangan

**Affiliations:** ^1^Laboratory of Molecular Biomedicine, Institute of Bioscience, Universiti Putra Malaysia, 43400 Serdang, Selangor, Malaysia; ^2^Department of Nutrition and Dietetics, Faculty of Medicine and Health Sciences, Universiti Putra Malaysia, 43400 Serdang, Selangor, Malaysia; ^3^UPM-MAKNA Cancer Research Laboratory, Institute of Bioscience, Universiti Putra Malaysia, 43400 Serdang, Selangor, Malaysia; ^4^Department of Pathology, Faculty of Medicine and Health Sciences, Universiti Putra Malaysia, 43400 Serdang, Selangor, Malaysia; ^5^Department of Biomedical Sciences, Faculty of Medicine and Health Sciences, Universiti Putra Malaysia, 43400 Serdang, Selangor, Malaysia

## Abstract

Nutritional or dietary factors have drawn attention due to their potential as an effective chemopreventive agent, which is considered a more rational strategy in cancer treatment. This study was designed to evaluate the effect of IP_6_ extracted from rice bran on azoxymethane- (AOM-) induced colorectal cancer (CRC) in rats. Initially, male Sprague Dawley rats were divided into 5 groups, with 6 rats in each group. The rats received two intraperitoneal (*i.p.*) injections of AOM in saline (15 mg/kg body weight) over a 2-week period to induce CRC. IP_6_ was given in three concentrations, 0.2% (w/v), 0.5% (w/v), and 1.0% (w/v), via drinking water for 16 weeks. The deregulation of the Wnt/**β**-catenin signaling pathway and the expression of cyclooxygenase (COX)-2 have been implicated in colorectal tumorigenesis. **β**-Catenin and COX-2 expressions were analysed using the quantitative RT-PCR and Western blotting. Herein, we reported that the administration of IP_6_ markedly suppressed the incidence of tumors when compared to the control. Interestingly, the administration of IP_6_ had also markedly decreased **β**-catenin and COX-2 in colon tumors. Thus, the downregulation of **β**-catenin and COX-2 could play a role in inhibiting the CRC development induced by IP_6_ and thereby act as a potent anticancer agent.

## 1. Introduction

Inositol hexaphosphate (IP_6_) is a dietary component that constitutes approximately 1 to 5% of the weight of most cereals, nuts, oil seeds, legumes, and grains [1, 2]. In particular, approximately 9.5 to 14.5% of the weight of rice bran is composed of IP_6_. Phytic acid or IP_6_ is a polyphosphorylated carbohydrate with six phosphate groups, each attached to a carbon atom [3]. *Myo*inositol is the parent compound of IP_6_. The phosphate grouping in positions 1, 2, and 3 (axial-equatorial-axial) is unique for IP_6_ and suggests tremendous chelating potential [3], thereby it has a potential role as anticancer agent. According to previous research, IP_6_ exhibits a variety of chemopreventive properties, including antioxidant and anticancer properties [4, 5]. Matejuk and Shamsuddin suggested that IP_6_ acts as a protective agent in cancer development [6].

Preclinical studies have been conducted to evaluate the potential anticancer properties and tolerability of IP_6_ in cancer treatment [6]. However, because these studies have not evaluated the anticancer potential and toxicity effects of IP_6_ extracted from rice bran, its specific mechanism of action as an anticancer agent remains unexplored. Furthermore, the antiproliferative activity of IP_6_ is most likely mediated through the coordinated activity of a number of molecules, although the exact signaling network involved in eliciting this response is unclear. Therefore, our study aims to evaluate the anticancer potential of IP_6_ extracted from rice bran and to determine its mechanism of actions. 

Furthermore, Singh et al. [7] have previously analyzed the anticancer efficacy of oral IP_6_ against human prostate carcinoma xenograft *in vivo*, in which IP_6_ suppressed the tumor growth without any toxicity. IP_6_ also has been found to be effective in animal tumorigenesis models of other cancer types without any toxicity [8]. In addition, our earlier study has reported that no toxic effect has been found in liver and kidney after the administration of IP_6_ to treat colorectal cancer (CRC) in AOM-induced rats [9]. 

CRC is ranked as the third most prevalent cancer and the second leading cause of disease-related deaths in the US [10]. CRC results in, when normal glandular epithelial cells accumulate acquired genetic and epigenetic changes that transform the cells into invasive adenocarcinomas. In other words, when normal epithelium transforms into an adenoma, it can eventually invade nearby colon tissue and metastasizes [11]. Carcinogenesis may be targeted at multiple points during its sequence to prevent the development of adenocarcinoma. Potential preventive agents that help suppress colon carcinogenesis can lead to chemoprevention of the disease [12]. To date, many chemopreventive agents from natural products and their phytochemical constituents are known to interact with diverse molecular targets during carcinogenesis [13]. A broad range of nutraceutical compounds possess remarkable therapeutic properties; therefore, we aimed to examine the development of an alternative compound from Malaysian sources, particularly rice bran. Rice bran which is composed of pericarp, seed coat, nucellus, aleurone layers, and germ is a byproduct of rice milling in the production of white rice.

Abnormal activation of the Wnt/*β*-catenin pathway has been implicated in human CRC [14]. This activation occurs due to the overexpression of the Wnt ligand and/or mutations in the downstream molecules of the Wnt signaling cascade, such as the *APC* and **β**-*catenin* genes [15]. Therefore, the Wnt/*β*-catenin pathway is considered a therapeutic target for the prevention and treatment of CRC.

Spychalski et al. [14] reported that, in addition to excessive *β*-catenin, overexpression of COX-2 and increased prostaglandin (PG) production from free arachidonic acid also contribute to CRC development. Overexpression of cyclooxygenase-2 (COX-2) has been reported in approximately 90% of colon tumors and premalignant colorectal adenomas [16, 17]. COX-2 is an inducible prostaglandin G/H synthase and was identified to have a vital function in prostaglandin (PG) synthesis. In addition to the COX-2/PGE2 signaling pathway, a lot of natural products and their active phytochemicals have been proven to have chemopreventive potential that can modulate various signal transduction pathways underlying colon carcinogenesis [13]. This was proven by Reddy et al. [18] who have shown that wheat bran fractions play a role in reducing iNOS and COX-2 expressions in CRC. Moreover, reduction in transcriptional activity of COX-2 has been shown after administration of anthocyanin-rich extracts of bilberry and grape in AOM-induced rat [19]. Bakhle [20] has previously reported that the COX-2 protein is normally absent in cells. To the best of our knowledge, the effects of IP_6_ in altering the expression of *β*-catenin and COX-2 have not been described earlier. Hence, we intend to investigate the effects of IP_6_ for the first time on these two pathways during colon tumorigenesis.

## 2. Materials and Methods

### 2.1. Chemicals

Hexane and hydrochloric acid (HCl) were purchased from Merck (Darmstadt, Germany). Iron chloride (FeCl_3_), azoxymethane, and sodium hydroxide (NaOH) were purchased from Sigma (St. Louis, MO, USA). Western blotting reagents were purchased from Bio-RAD (CA, USA). Dimethylformamide (DMF) was purchased from Fermentas (Lithuania, EU). Other chemicals used in this study were of the highest commercial grade.

### 2.2. Preparation of IP_6_

Freshly milled raw rice bran samples from mixed local varieties (MR 84 and MR 219) were kindly supplied by the BERNAS Milling Plant (Selangor, Malaysia). The bran was microwaved immediately and stabilized according to the method used by Ramezanzadeh et al. [21]. Then, 2 mL of hexane was added to every 0.5 g of rice bran and soaked overnight to produce defatted rice bran [22]. The rice bran was then filtered and dried by evaporation using a vacuum pump apparatus. After that, IP_6_ was extracted as follows: using a slight modification to previously described methods [23]. The defatted rice bran was combined with hydrochloric acid (0.5 M HCl) and continuously shaken in an orbital mixer for 2 hours at room temperature. The mixture was then centrifuged at 17,300 g for 30 minutes at 15°C, and the supernatant containing IP_6_ was collected. To neutralize the sample, FeCl_3_ was added to the IP_6_, and the mixture was placed in a boiling water bath to allow complete precipitation of the ferric phytate complexes. Ferric phytate was then heated with NaOH and centrifuged to obtain ferum (III), hydroxide (pellet), and sodium phytate (supernatant) [24]. Finally, the supernatant was collected and freeze dried. We successfully extracted and obtained the pure IP_6_ from rice bran in our laboratory. The determination of IP_6_ was done by HPLC analysis [23].

### 2.3. Animals

Male Sprague Dawley rats weighing approximately 50 g were acclimatized for seven days and fed on normal rat chow, according to the AIN 76-A guidelines, and tap water was provided *ad libitum*. Rats were housed in individual cages, which were fully ventilated and maintained at room temperature on a 12-hour light-dark cycle. All of the experimental protocols involving animals were approved by the Animal Care and Use Committee of the Faculty of Medicine and Health Sciences, Universiti Putra Malaysia (UPM), Serdang, Selangor, Malaysia.

### 2.4. Experimental Design

Rats were acclimatized for seven days and randomized into experimental and control groups. The animals were injected intraperitoneally with azoxymethane (AOM), a specific carcinogen that was diluted in saline, once per week (15 mg/kg body weight), over a 2-week period to induce colonic tumors [9, 25]. Rats were divided into 5 groups (*n* = 6). Group 1 was the control group. Groups 2–5 received intraperitoneal (*i.p.*) injections of AOM once per week, for two consecutive weeks at a dose of 15 mg/kg body weight, as mentioned above. Starting one week after the second dosing with AOM, groups 3–5 received various concentrations of IP_6_ [0.2% (w/v), 0.5% (w/v), and 1.0% (w/v)] in their drinking water for a period of 16 weeks. These concentrations were achievable physiologically in rat tumor models considering the doses used which give rise to inhibition of tumor incidence [9, 26]. All of the rats were carefully observed on a daily basis, and their body weights were recorded weekly. At the end of the experiment, all of the rats were euthanized by cervical decapitation, and the whole colon tissues were collected, weighed, and processed immediately for further analysis. 

### 2.5. Detection of Aberrant Crypt Foci (ACF) and Tumor Assessment

The colons were resected and flushed with 10% neutralized formalin to remove residual bowel contents, cut open longitudinally and submerged overnight in 10% (v/v) neutralized formalin. The tissue samples were dehydrated in an ascending series of alcohol dehydration, cleared with xylene, and wax impregnated with paraffin wax for 14 hours in an automatic tissue processor machine. Sections (3 *μ*M) were stained with hematoxylin and eosin (H & E).

 ACF were identified using a published set of criteria which distinguishes these lesions from normal crypts [25]. Tumor assessment parameters have been previously described by Bird [25]. Briefly, tumor incidence is the percentage of total animals with tumors as follows:

(1) tumor  incidence=total  animals  with  adenoma  and/or  adenocarcinomatotal  animals.

### 2.6. Analysis of *β*-Catenin and COX-2 Expression by Real-Time PCR

The extraction of total RNA from fresh whole colon tumor tissue was performed using TRI Reagent (Sigma, MO, USA). Tissue disruption and homogenization were performed such that the colon tumor tissue was weighed out (20–30 mg), immediately placed in liquid nitrogen, and homogenized using a tissue grinder. The tissue powder and liquid nitrogen were decanted into an RNase-free, 2 mL microcentrifuge tube, and the liquid nitrogen was allowed to evaporate. Then, the tissue samples were homogenized in 1 mL of TRI Reagent per 50–100 mg of tissue for 10 minutes. RNA extraction was then performed according to the manufacturer's instructions, and 1 *μ*g of the total RNA sample was reverse transcribed using the GeneAmp Gold RNA PCR Core Kit (Applied Biosystems, California, USA) in a final reaction volume of 20 *μ*L, according to the manufacturer's protocol. The reverse transcription reaction was carried out at 42°C for 30 minutes in an authorized thermal cycler (Eppendorf, NY, USA), followed by a 10-minute step at 99°C to denature the enzyme; then, it was cooled to 4°C. This cDNA was then used as a template for amplification in real-time PCR reactions. Quantitative real-time PCR was performed in a reaction volume of 20 *μ*L, according to the manufacturer's instructions for the KAPA SYBR Green FAST qPCR kits (Kapa Biosystems, Boston, MA, USA). Nucleotides primer sequences of rat origin were used (Table 1). Briefly, 18.2 *μ*L of master mix, 0.8 *μ*L of primer assay (10x), and 1 *μ*L of template cDNA (20 ng) were added to each well. After a brief centrifugation, the PCR plate was subjected to 40 cycles of the following conditions: (i) PCR activation at 95°C for 20 seconds, (ii) denaturation at 95°C for 3 seconds, and (iii) annealing/extension at 60°C for 20 seconds. All samples and controls were run in triplicate on a Mastercycler realplex system (Eppendorf, NY, USA). The quantitative RT PCR data were analyzed using a comparative threshold (Ct) method, and the fold inductions of the samples were compared with the untreated samples. *β*-actin was used as an internal reference gene to normalize the expression of the target genes.

### 2.7. Western Blot Analyses of *β*-Catenin and COX-2

The fresh colon tumor tissue disruption and homogenization steps for extracting proteins were similar to those described for total RNA extraction. The samples were homogenized in Buffer RLT using a QIAshredder homogenizer. Protein extraction was performed using the AllPrep DNA/RNA/Protein Mini Kit, according to the manufacturer's protocol (Qiagen, Duesseldorf, Germany). Next, the protein concentration was determined by the Bradford assay, according to the manufacturer's protocol (Bio-RAD, CA, USA). The protein (50 *μ*g) was separated by 12% sodium dodecyl sulfate polyacrylamide gel electrophoresis (SDS-PAGE) and transferred onto a piece of PVDF membrane using transfer buffer (25 mM Tris base, 190 mM glycine, and 20% (v/v) methanol, pH 8.3). After transfer, the PVDF membrane was blocked at room temperature with blocking solution (25 mM Tris base, 0.3 M NaCl, and 5% milk diluent) (Bio-RAD, CA, USA) for 30 minutes. The membrane was then incubated overnight with primary antibodies at a 1 : 1000 dilution (*β*-catenin rabbit monoclonal antibody and COX-2 rabbit monoclonal antibody from Santa Cruz Biotechnology, CA, USA), followed by a 1-hour incubation with alkaline phosphatase-labeled goat anti-mouse secondary antibody (Santa Cruz Biotechnology, TX, USA) at a 1 : 10000 dilution in Tris-buffered saline (TBS) and 0.5% Tween 20. Immunodetection was performed by the addition of a developing solution. The developing solution for alkaline phosphatase conjugated antibodies consisted of 10 mL alkaline phosphatase buffer (100 mM Tris HCl, 100 mM NaCl, and 5 mM MgCl_2_; pH 9.5), 33 *μ*L BCIP (0.5 g 5-bromo-4-chloro-3-indolylphosphate-p-toluidine salt (Fermentas, Lithuania, EU) in 10 mL of 100% (v/v) dimethylformamide (DMF)), and 66 *μ*L NBT (0.75 g nitroblue tetrazolium chloride (Fermentas, Lithuania, EU) in 10 mL of 70% (v/v) DMF). The reaction was stopped when the desired protein band appeared. Densitometric analysis of band intensities obtained from western blotting experiments were carried out using ImageJ software (National Institutes of Health (NIH), USA).

### 2.8. Statistical Analysis

Data were expressed as the mean ± standard deviation (SD) and statistically analyzed using SPSS version 10, with a one-way ANOVA with Tukey's test and a significance level of *P* < 0.05.

## 3. Results

### 3.1. Identification of IP_6_

IP_6_ was analyzed by reversed-phase high-performance liquid chromatography (HPLC). HPLC chromatograms were compared with those of standard phytic acid. As we reported earlier [9, 23], based on the HPLC chromatogram data, two peaks were represented as pure inositol hexaphosphate.

### 3.2. Body Weight

**Figure 1:**
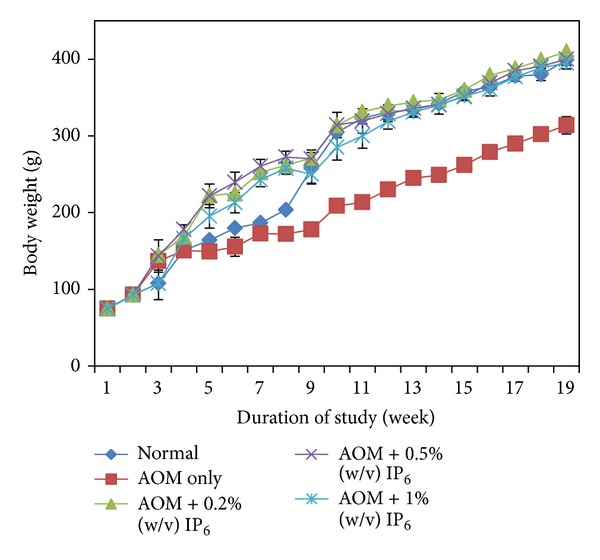
General observation on body weight of experimental rats throughout the study. IP_6_ increases the body weight in all IP_6_-fed groups relative to the normal group. Groups are normal, AOM only, AOM + 0.2% (w/v) of IP_6_, AOM + 0.5% (w/v) of IP_6_, and AOM + 1% (w/v) of IP_6_. (AOM = azoxymethane, IP_6_ = inositol hexaphosphate).

Figure 1 represents the changes in the rat body weights during the experiment. Body weights were compared between all groups (normal, AOM only, AOM + 0.2% (w/v) of IP_6_, AOM + 0.5% (w/v) of IP_6_, and AOM + 1% (w/v) of IP_6_). The plotted graph in Figure 1 shows that the body weights of all of the rats increased over time. When compared with the normal group, the IP_6_-fed groups (AOM + 0.2% (w/v) of IP_6_, AOM + 0.5% (w/v) of IP_6_, and AOM + 1% (w/v) of IP_6_) showed no significant differences in body weight, even after 16 weeks of IP_6_ treatment. However, there was a significant (*P* < 0.05) difference between the body weight of the positive control group (AOM only) and the body weight of the rats in the IP_6_-fed and normal groups. In addition, the body weight of the AOM-only group was significantly lower than that of the IP_6_-fed and normal groups; these results support the expectation that exposure to the carcinogen AOM would cause weight loss. Additionally, administration of IP_6_ during the early treatment may boost growth (Figure 1). However, the mechanism is unknown. These data clearly showed that daily consumption of IP_6_ even for longer duration of treatment was not toxic to normal rats and also maintained or even increased the body weight of AOM-treated rats versus normal rats, potentially inhibiting abnormal growths in the colon, which were further examined by histopathological analyses.

### 3.3. Incidence of ACF and Tumors

**Figure 2:**
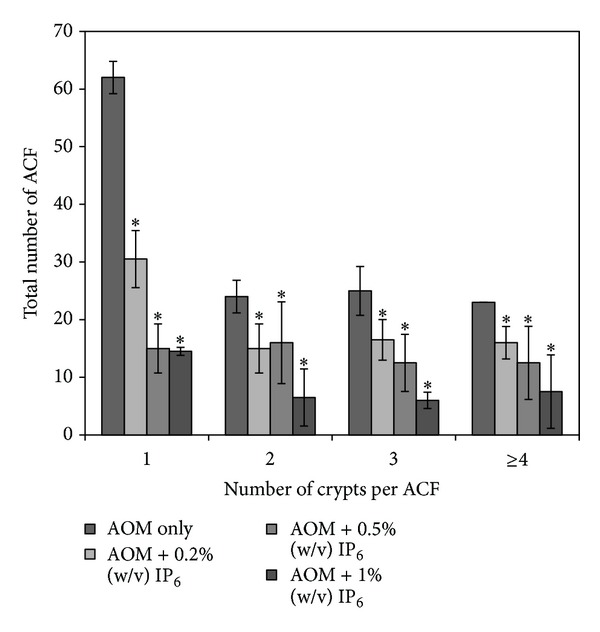
Effect of IP_6_ on AOM-induced colonic aberrant crypt foci (ACF) in rats. IP_6_ decreases the number of ACF in all IP_6_-fed groups relative to the AOM-only group. Each value expressed as mean ± SD (*n* = 6). *Indicates significant difference by Tukey's test (*P* < 0.05). Groups are AOM only, AOM + 0.2% (w/v) of IP_6_, AOM + 0.5% (w/v) of IP_6_, and AOM + 1% (w/v) of IP_6_. (AOM = azoxymethane, ACF = aberrant crypt foci, IP_6_ = inositol hexaphosphate).

**Figure 3:**
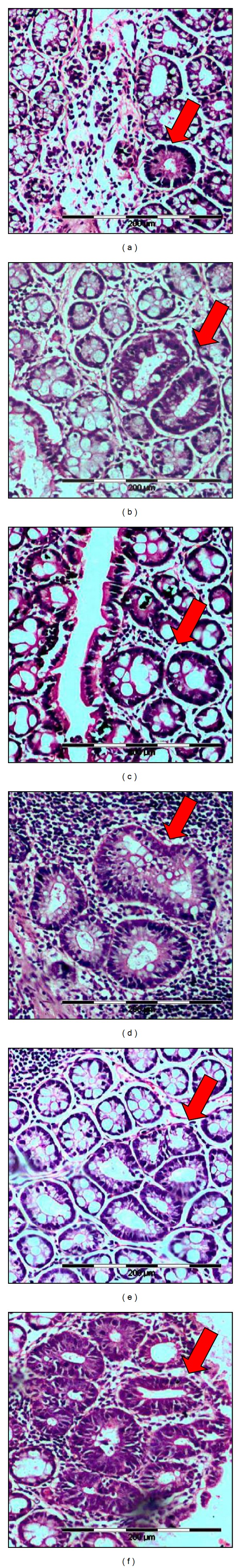
Histological analysis of aberrant crypt foci (ACF) (magnification, 400x). (a) small ACF containing 1 crypt, (b) small ACF containing 2 crypts, (c) medium ACF containing 3 crypts, and (d, e, f) large ACF containing more than 4 crypts. (ACF = aberrant crypt foci).

ACF is a preneoplastic lesion which can be characterized by clusters of mucosal cells with an enlarged and thicker layer of epithelia compared to the surrounding normal crypts. ACF may progress into polyps, later develop into adenomas, and eventually become invasive carcinomas [27]. Although not all ACF may develop into cancer, but all colon cancers arise from ACF as evidenced by several studies [27]. Jen et al. [28] proposed that only dysplastic ACF progress to adenoma and adenocarcinoma. The beneficial effect of various dosages of phytic acid (IP_6_) (0.2% (w/v), 0.5% (w/v), and 1% (w/v)) on the reduction of ACF development was further demonstrated. As shown in Figure 2, the analysis of ACF formation incidence demonstrated that the administration of rice bran IP_6_ significantly reduced the total number of ACF (*P* < 0.05) in all IP_6_-fed groups in comparison with the untreated rats and AOM-only group, thereby leading to the inhibition of ACF formation which may or may not develop into the invasive carcinoma.  Figure 3 summarized the histological analysis of aberrant crypt foci (ACF) with surrounding normal tissue using H & E staining. The images (Figure 3) represented the incidence of ACF taken from group of rats induced with AOM only (without treatments of IP_6_).

**Figure 4:**
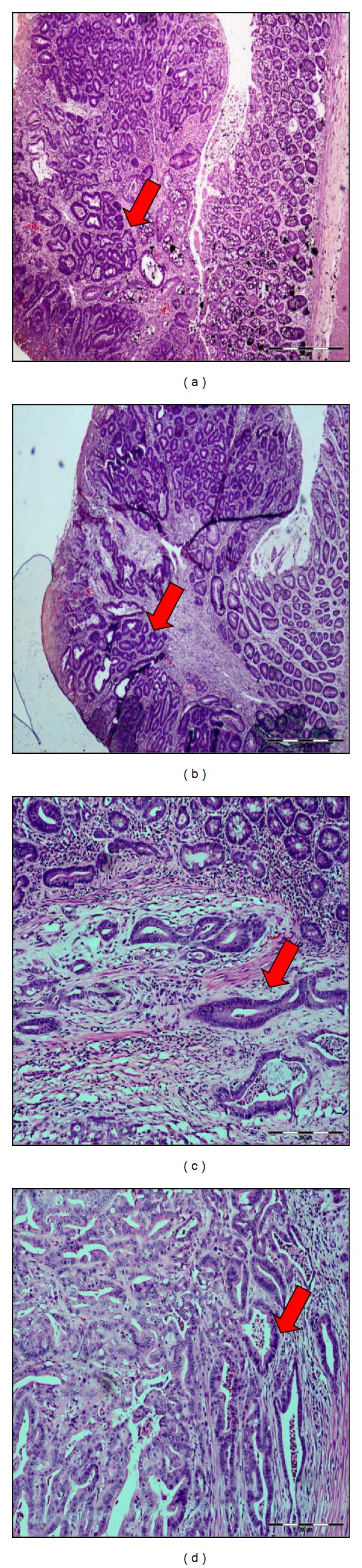
Histopathology of colonic lesions developed in rats' group treated with AOM only. (a) Adenomatous polyps originated from the colon of a few rats (a and b). The polyps are located in the mucosal layer only. (b) Adenocarcinoma (c and d) showing the invasive gland in the submucosa and muscular layer (magnification 400x).

An adenoma is defined as a benign tumor of glandular origin. Colonic adenoma consists of proliferation on colonic glands lined by neoplastic colonic epithelium, while an adenocarcinoma is a malignant tumor originating in glandular epithelium. Colonic adenocarcinoma is composed of invasive glands lined by pleomorphism and hyperchromatic epithelium.  Figure 4 showed the histopathology of colonic lesions developed by AOM-induced CRC in rats. The images (Figure 4) represented the incidence of adenoma and adenocarcinoma taken from rats induced with AOM only (without treatments of IP_6_).

The incidence of adenoma, adenocarcinoma, and total tumors (adenoma + adenocarcinoma) in different study groups is summarized in Table 2. The highest incidence of adenoma, adenocarcinoma, and total tumors occurred in the AOM-only group. However, administration with IP_6_ (0.2, 0.5, and 1% (w/v)) significantly reduced the incidence of tumors in a dose-dependent manner when compared with the results of the AOM-only group (no IP_6_ treatment) (*P* < 0.05). None of the rats in the normal group developed colon tumor. Additionally, not all rats consist of both adenoma and adenocarcinoma, namely, total tumors (Table 2), and in accordance with our previous study which demonstrated that both adenoma and adenocarcinoma are developed in some of the rats [9]. Altogether, the administration of IP_6_ appeared to reduce the incidence of adenoma, adenocarcinoma, and total tumors.

### 3.4. IP_6_ Downregulated the mRNA Levels of *β*-Catenin and COX-2

**Figure 5:**
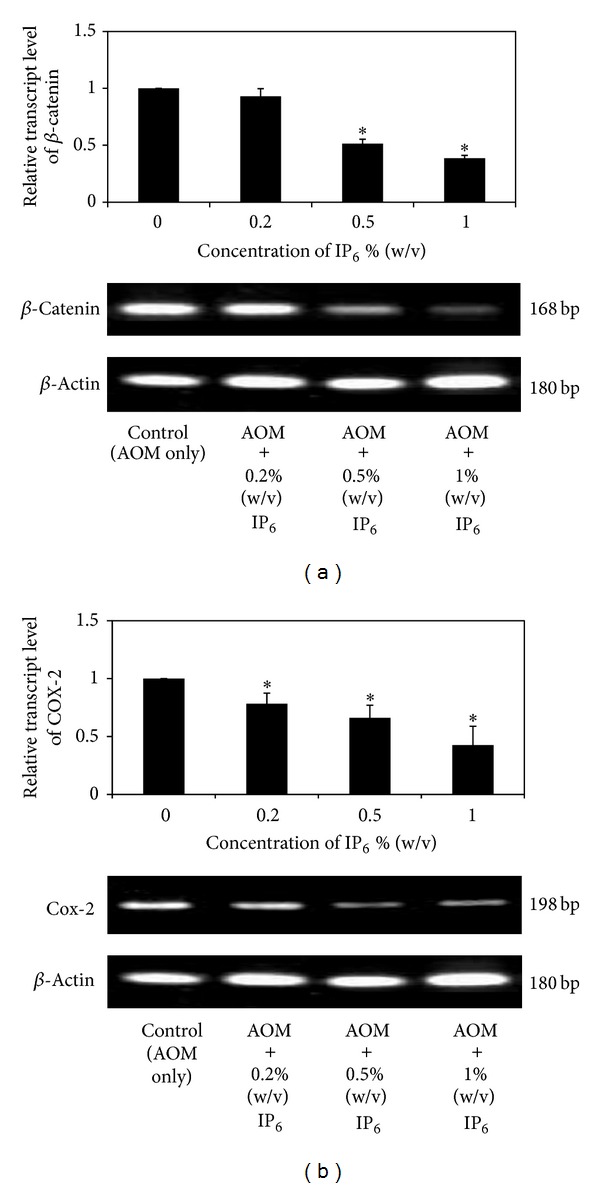
Expression of *β*-catenin and COX-2 at the mRNA level in rats with AOM-induced colon carcinogenesis after treatment with IP_6_. The expressions of *β*-catenin and COX-2 mRNA were assessed by quantitative RT PCR. IP_6_ downregulates the expression of (a) *β*-catenin and (b) COX-2 in a dose-dependent manner. The results show a typical pattern from the 3 experiments performed. *Indicates significant difference by Tukey's test (*P* < 0.05) relative to their respective control.

Our study successfully demonstrates that inhibition of tumor growth in AOM-induced rat CRC using IP_6_ treatment is accompanied by the downregulation of *β*-catenin.  Figure 5(a) shows that, compared with the controls, IP_6_-treated rats expressed less *β*-catenin at the mRNA level, especially for rats treated with the highest concentration of IP_6_, 1% (w/v). After treatment with 0.2%, 0.5%, and 1% (w/v) of IP_6_, *β*-catenin was significantly downregulated by 0.93-, 0.514-, and 0.385-fold, respectively, demonstrating a dose-dependent relationship (*P* < 0.05). 

Moreover, based on the fold changes relative to the control, IP_6_ also significantly inhibited the expression of COX-2 in AOM-induced rat colon carcinogenesis (*P* < 0.05). As shown in Figure 5(b), after treatment with IP_6_ (0.2%, 0.5%, and 1% (w/v)), COX-2 mRNA expression was significantly downregulated by 0.783-, 0.661-, and 0.426-fold, showing a dose-dependent relationship (*P* < 0.05).

### 3.5. IP_6_ Reduced the Expressions of *β*-Catenin and COX-2

**Figure 6:**
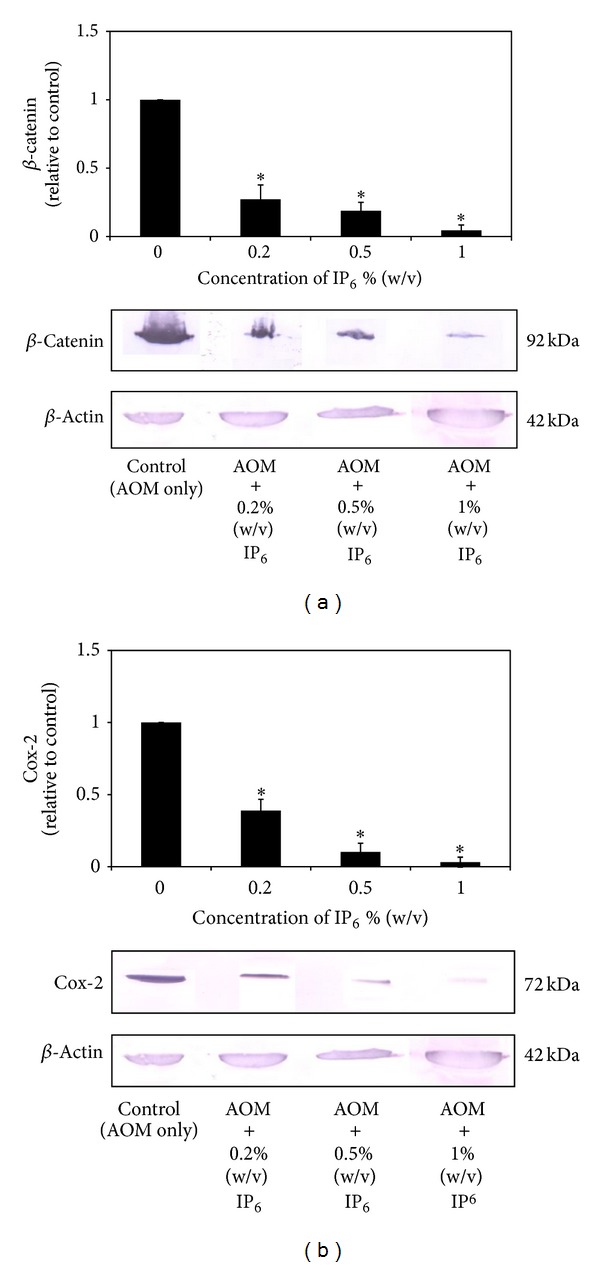
The effect of IP_6_ on the protein levels of *β*-catenin and COX-2 in rats with AOM-induced colon carcinogenesis. IP_6_ decreases the protein expression of *β*-catenin (a) and COX-2 (b) in a dose-dependent manner. Protein expression was normalized to the *β*-actin control. The results show a typical pattern from the 3 experiments performed. *Indicates significant difference by Tukey's test (*P* < 0.05) relative to their respective control.

The expression levels of *β*-catenin and COX-2 proteins for the control and experimental group of rats are presented in Figure 6. The results from the Western blot show that *β*-catenin and COX-2 were both overexpressed in the AOM-induced rats. As shown in Figure 6(a), *β*-catenin was detected as a 92-kDa band in AOM-induced rat colon carcinogenesis. Significant reduction in *β*-catenin protein expression was observed after treatment with IP_6_ (0.2%, 0.5%, and 1%) by 0.272-, 0.189-, and 0.045-fold relative to the control in a dose-dependent manner (*P* < 0.05). 

Furthermore, as shown in Figure 6(b), the immunoreactive bands of COX-2 were detected at 72 kDa (the molecular weight of COX-2) in AOM-induced rats. Similarly, because COX-2 expression was inhibited by IP_6_ at the mRNA level, it was also downregulated at the protein level. COX-2 protein expression in AOM-induced rat colon carcinogenesis was significantly decreased by 0.38-, 0.102-, and 0.031-fold after treatment with 0.2%, 0.5%, and 1% (w/v) of IP_6_, respectively, compared with the control group, thus showing a dose-dependent relationship (*P* < 0.05). All together, these results showed that the regulation or stability of *β*-catenin and COX-2 protein levels should be affected by IP_6_.

## 4. Discussion

The primary parameters used in cancer chemoprevention studies to assess the toxicity of a test compound include body weight gain profiles. Accordingly, changes in animal body weight, as a result of IP_6_ administration during the 16-week study period, were assessed to evaluate potential adverse health effects of the treatment. As mentioned earlier, all of the IP_6_-fed groups experienced body weight gain that was similar to the normal group and, therefore, did not show any significant differences. These results were also observed by Norazalina et al. [9] who previously studied the anticancer efficacy of AOM-induced CRC in rats; their results included the body weights of the rats that were treated with IP_6_ from rice bran. Similar findings were observed in the suppression of prostate cancer growth for IP_6_-fed mice [26]. 

A significant reduction in the body weight of AOM-induced group was observed when compared with the other groups; the body weights observed for the normal and IP_6_-fed groups were reliable because, according to Alfin-Slater and Kritchevsky [29], the weight loss that accompanies tumor development is thought to be associated with a reduction of not only body fat stores but also body mass. There is increasing evidence that the tumor directly affects the host's protein metabolism. Specifically, the protein turnover process is altered in the cancer-bearing host, and there is a net loss of nitrogen from nonmalignant tissues because protein degradation exceeds protein synthesis. Moreover, it is interesting to highlight that in all of the IP_6_-fed groups, even though all of the rats were initially treated with the carcinogen (AOM), their body weights showed no significant difference compared with that of the normal rats after 16 weeks of continuous IP_6_ treatment. Thus, the administration of IP_6_ appears to have an inverse effect on the reduction in body weight seen with AOM alone. However, the mechanism behind this effect is unknown.

The current data shows that the administration of IP_6_ in drinking water suppressed the number of ACF and thereby leading to the inhibition of tumor incidence in the colons of the IP_6_-fed groups as compared with the AOM-induced group. IP_6_ has been shown to reduce the number and size of colon adenomas, which are the precursor lesions of CRC. Our study suggests that IP_6_ extracted from rice bran might reduce the formation of tumors in the colon. The tumor inhibition action of IP_6_ has been supported by previous studies, which showed that the addition of IP_6_ to drinking water had a chemopreventive effect on CRC [9, 30, 31]. Furthermore, our earlier studies have shown the potential antiproliferative effects of IP_6_ from rice bran on CRC and hepatic cancer *in vitro *[32, 33].

Alterations in the *APC* or **β*-catenin* gene are regarded as early critical events during CRC and are therefore considered to play a gate keeper role in the development of CRC in both humans and preclinical models [34–36]. Mutations in the *APC* or **β*-catenin* gene were proved to repress the degradation of the protein and generate *β*-catenin accumulations in the cytosol [37, 38]. The excessive *β*-catenin functions as a transcriptional activator when complexed with members of the T-cell factor (TCF) family of DNA binding proteins [39, 40]. Furthermore, target genes of **β*-catenin* signaling pathway, such as *c-myc* and *cyclin D1*, are growth-promoting genes, suggesting that this pathway is potentially an oncogenic pathway [41, 42]. Expression of non-p-*β*-catenin was elevated in AOM-induced experimental CRC [43], also colon adenocarcinoma cells [44]. Other natural compounds have the ability to reduce the expression of non-p-*β*-catenin during colon tumorigenesis [43–45]. Our present study further supported previous reports on the expression of *β*-catenin which was increased while induction with AOM [46]. We assumed that IP_6_ might be involved in regulation or stability of *β*-catenin level in CRC as a previous study has also reported the role of inositol polyphosphates in Wnt signaling pathway, by which IP_5_ accumulates in response to Wnt3a, thereby playing an essential component of signaling of the canonical pathway to the level of *β*-catenin accumulation [47].

Interestingly, we demonstrated that the inhibition of CRC by IP_6_ may be mediated through inactivation of *COX-2* gene. The results showed a dose-dependent reduction in the level of COX-2 in IP_6_-treated rats with AOM-induced CRC. Although the mechanism underlying how IP_6_ modulates the expression of the COX-2 is still unknown, it is likely that the downregulation of COX-2 might be the cause of repressed tumor growth and subsequent tumor cell death. The current data indicate that oral administration of IP_6_ was associated with a decreased incidence of CRC through reductions in the number and size of adenomas and invasive carcinomas. These observations suggest the protective effects of IP_6_ against AOM-induced colon cancer in animal models. 

Our data demonstrate that the anticancer action of IP_6_ occurs via a reduction in tumor incidence, which subsequently leads to tumor suppression and the depletion of COX-2 and *β*-catenin expressions, whereby all of these are implicated in AOM-induced colon carcinogenesis. These data further support previous studies suggesting that IP_6_ acts as an anticancer agent by reversing the proliferative effects of carcinogens [2].

Interestingly, several studies have shown that COX-2 may be regulated by the Wnt/*β*-catenin signaling pathway [48–50]. It seems likely that IP_6_ may downmodulate Wnt signaling via the inhibition of *β*-catenin, thus reducing levels of COX-2. This effect was shown by a marked inhibition of COX-2 expression after treatment with IP_6_. Real-time PCR determination of the COX-2 mRNA level in IP_6_-treated rats with AOM-induced CRC indicates that COX-2 is significantly downregulated; this effect correlates with the observed protein levels as well. Therefore, COX-2 expression may be suppressed via inhibition of Wnt/*β*-catenin signaling. The pharmacological inhibition of COX-2 may therefore provide an effective chemoprotective method against CRC. To further support this finding and previous studies that correlate *β*-catenin with COX-2, we suggest that the underlying inhibitory mechanism of IP_6_ occurs via the suppression of *β*-catenin expression through COX-2 regulation or vice versa. This correlation at the mRNA and protein levels strongly supports the reduction of intestinal neoplasia by *β*-catenin and COX-2 inhibitions, suggesting that IP_6_ may play a therapeutic role in CRC and may contribute to new strategies in the prevention and treatment of this disease.

## 5. Conclusion

The observed modulatory influences of rice bran IP_6_ at the level of cell proliferation, by inhibiting *β*-catenin and COX-2 during AOM-induced CRC, have not been noted previously and suggest that the intake of rice bran IP_6_ may be used in future clinical chemopreventive trials to monitor the responsiveness of the CRC development to the chemopreventive agents. 

## Figures and Tables

**Table 1:** Nucleotide sequence for PCR primers for amplification and sequence-specific detection.

Pair no.	Primer name	Accession no.	Oligonucleotides (5′-3′) sequence	Product size (bp)
1	*β*-catenin	NM-001165902	F-ACAGCACCTTCAGCACTCT R-AAGTTCTTGGCTATTACGACA	168
2	COX-2	L25925	F-ACAGGAGAGAAAGAAATGGCTGCAGAGT R-CAGTATTGAGGAGAACAGATGGGATT	198
3	*β*-actin	NM-031144	F-TCACCCACACTGTGCCCATCTATGA R-GTCACGCACGATTTCCCTCTCAGC	180

**Table 2:** Incidence of tumor of the IP_6_-treated rats with AOM-induced colon carcinogenesis.

Group no.	Treatment	No. of rats	Incidence (%) of adenoma and/or adenocarcinoma		
			Adenoma	Adenocarcinoma	Total
1	Normal	6	0	0	0
2	AOM only	6	4/6 (67%)^a^	5/6 (83%)^b^	5/6 (83%)^b^
3	AOM + 0.2% (w/v) IP_6_	6	3/6 (50%)^c^	3/6 (50%)^c^	4/6 (67%)^d^
4	AOM + 0.5% (w/v) IP_6_	6	1/6 (17%)^e^	2/6 (33%)^f^	3/6 (50%)^g^
5	AOM + 1% (w/v) IP_6_	6	1/6 (17%)^e^	1/6 (17%)^e^	2/6 (33%)^f^

Groups: normal, AOM only, AOM + 0.2% (w/v) of IP_6_, AOM + 0.5% (w/v) of IP_6_, AOM + 1% (w/v) of IP_6_, and (IP_6_: inositol hexaphosphate, AOM: azoxymethane). Values in the same column with different superscript indicate significant difference by the Tukey's test (*P* < 0.05). Adenoma is defined as a benign tumor of glandular origin. Adenocarcinoma is a malignant tumor originating in glandular epithelium. Total tumor consists of both adenoma and adenocarcinoma in the colon tumor tissue.
